# Metabolic Evidence Rather Than Amounts of Red or Processed Meat as a Risk on Korean Colorectal Cancer

**DOI:** 10.3390/metabo11070462

**Published:** 2021-07-16

**Authors:** Eunbee Kim, Joon Seok Lee, Eunjae Kim, Myung-Ah Lee, Alfred N. Fonteh, Michael Kwong, Yoon Hee Cho, Un Jae Lee, Mihi Yang

**Affiliations:** 1 Department of Toxicology, College of Pharmacy, Sookmyung Women’s University, Seoul 04310, Korea; keb940417@naver.com; 2 Agilent Technologies Korea, Seoul 06621, Korea; joon-seok.lee@agilent.com; 3 College of Pharmacy, Massachusetts College of Pharmacy and Health Sciences, Worcester, MA 02115, USA; ekim2@stu.mcphs.edu; 4 Department of Internal Medicine, Seoul St. Mary’s Hospital, Cancer Research Institute, College of Medicine, The Catholic University of Korea, Seoul 06591, Korea; angelamd@catholic.ac.kr; 5 Molecular Neurology Program, Huntington Medical Research Institutes, Pasadena, CA 91105, USA; alfred.fonteh@hmri.org (A.N.F.); micgkwong@gmail.com (M.K.); 6 Department of Biomedical and Pharmaceutical Sciences, The University of Montana, Missoula, MT 59812, USA; unicho3@gmail.com; 7 Goodbeing Co., Ltd., Seoul 04310, Korea; unjae@hotmail.com

**Keywords:** colorectal cancer, red meat, processed meat, heterocyclic amines, lipid

## Abstract

The incidence of colorectal cancer (CRC) has increased in Korea, a newly-*industrialized* Asian country, with the dramatic increase of meat intake. To assess the risks of red or processed meat consumption on CRC, we performed a case-control study with biological monitoring of urinary1-OHP, PhIP, and MeIQx for the meat exposure; dG-C8 MeIQx and dG-C8 PhIP for HCA-induced DNA adducts; and homocysteine and C-reactive protein (CRP) in blood as well as malondialdehyde (MDA) and 31fatty acids in urine for inflammation and lipid alteration. We further analyzed global DNA methylation and expression of 15 CRC-related genes. As a result, the consumption of red or processed meat was not higher in the cases than in the controls. However, urinary MeIQx and PhIP were associated with the intake of red meat and urinary 1-OHP. MDA and multiple fatty acids were related to the exposure biomarkers. Most of the 31 fatty acids and multiple saturated fatty acids were higher in the cases than in the controls. Finally, the cases showed upregulation of *PTGS2*, which is related to pro-inflammatory fatty acids. This study describes indirect mechanisms of CRC via lipid alteration with a series of processes including exposure to red meat, alteration of fatty acids, and relevant gene expression.

## 1. Introduction

The incidence of colorectal cancer (CRC) has increased worldwide. The World Health Organization (WHO) and International Agency for Research on Cancer (IARC) [1] recently declared that over-intake of red meat and/or processed meat increases CRC risk, based on 10 cohorts and a meta-analysis [1]. A recent Global Health Data Exchange review indicated that red and processed meat intake accounts for 1.77% and 1.18%, respectively, of worldwide mortality of CRC [2]. Therefore, red and processed meat intake has been emphasized as one of the major avoidable causes of CRC. In particular, Korea, a newly-*industrialized* Asian country, showed a more than two-fold increase in the incidence of CRC over the last two decades, i.e., from approximately 20 to 43 cases per 100,000 persons [3]. Rapid industrialization has brought dramatic changes in Korean lifestyles and patterns of food consumption toward a more Western diet. This change in diet has been suspected as one of the main causes for the increased incidence of CRC in Korea. For example, Korean meat consumption dramatically increased during 1970–2005, from 10.4 g to 75 g/day [4]. The 5-year prevalence and age-standardized incidence of CRC were even higher in Korea and Japan than those reported in Western countries [5,6]. 

Many researchers have proposed various initiators, such as heme iron [7], nitrites, and chemicals produced by cooking or processing, e.g., polycyclic aromatic hydrocarbons (PAHs) or heterocyclic amines (HCAs) [8] as the carcinogenic mechanisms of red and processed meats on CRC. DNA-adducts of these chemicals can explain the toxic mechanisms related to red and processed meat exposure [9], and compensate for the drawbacks of case-control studies for longitudinal exposure. In addition, the involvement of red meat in sporadic CRC or colitis-associated cancer is also evidenced by the altered expression of various genes, such as *PTGS2, APC, KRAS,* and *P53* [10]. Epigenetic modification, such as *MLH1* hypermethylation that results in a loss of mismatch repair methylation change, has been suggested as a longitudinal biomarker for CRC [11]. Moreover, lipid peroxidation and altered metabolites are currently considered a hallmark characteristic of CRC [12,13,14]. Therefore, genetic, epigenetic, and lipidic biomarkers are needed to provide new insights into CRC risk, development, and progression.

Hence, we aim to test whether and how red and processed meat intake affects CRC onset among the Korean population. WHO/IARC declared the risks of red and processed meat intake on CRC [1]; however, the risks are not clear, or are controversial in Asian populations [15]. Therefore, we performed a molecular epidemiological study to clarify the relationship and carcinogenic mechanisms between a cause, i.e., red and processed meat intake, and a disease, CRC. For this purpose, we measured red and processed meat intake using a food frequency questionnaire (FFQ) among CRC cases and age- and sex-matched controls, and quantified urinary metabolites of polycyclic aromatic hydrocarbons (PAHs) and heterocyclic amines (HCAs) from red or processed meat. We selected 1-hydroxoxypyrene (1-OHP) for assessment of PAHs [16]; 2-Amino-3,8-dimethylimidazo[4,5-f] quinoxaline (MeIQx) or 2-Amino-1-methyl-6-phenylimidazo[4,5-b]pyridine (PhIP) for HCAs [8]; deoxyguanosine (dG)-C8 MeIQx and dG-C8 PhIP for measurement of HCA- DNA adducts [17]; and various responsive biomarkers for CRC, including expression differences of 15 CRC-related genes, i.e., *PTGS2, APC, KRAS,* etc., [10,11,18,19,20,21], global DNA methylation levels [22], lipid alterations in urinary 31 fatty acids [23], levels of lipid peroxidation with urinary malondialdehyde (MDA) [24], and inflammation status with homocysteine [25] and C-reactive protein (CRP) [26] in blood.

## 2. Results

### 2.1. Characteristics of Subjects

Table 1 shows the general characteristics of all subjects, including levels of meat and lipid intakes. Red meat and lipid consumption did not differ between the cases and controls, while the control group even consumed more processed meat than the cases (Table 1). The cases were newly diagnosed with CRC; thus, most were at early stages of the disease, and most locations of CRC were distal (Table S1). We also studied some confounders for the exposure biomarkers and metabolites, e.g., tobacco smoking and fish intake, which may induce HCAs [27] and ω-3 fatty acids [28], respectively. These two factors were not different between the two groups (Table 1).

### 2.2. Measurement of Exposure and Response Biomarkers

Measurements of exposure and response biomarkers are shown in Table 2. To assess PAHs and HCAs attributable to red and processed meat intake, we measured urinary 1-OHP for PAHs and urinary PhIP and MeIQx for HCAs. The ranges of urinary 1-OHP, PhIP, and MeIQx levels were 0.04–0. 33 μg/L (median, 0.14), 0.06–38.25 ng/L (median, 3.44), and 3.53–99.32 ng/L (median, 13.51), respectively (limit of quantification, LOQ, 0.08 μg/L, 0.13 ng/L, and 0.8 ng/L, respectively).

To assess PAHs- and/or HCAs-induced DNA adducts, the levels of dG-C8- MeIQx and dG-C8- PhIP were measured. The levels of dG-C8- MeIQx followed the normal distribution (range, 2.86–3.97 μg/L/μg of DNA; *p* = 0.34), while dG-C8- PhIP was not detected in any of the subjects (LOQs of dG-C8-MeIQx and -PhIP, 0.16 μg/L/μg of DNA).

To assess lipid peroxidation and inflammation status in response to CRC, urinary MDA and homocysteine and CRP in blood were measured. The ranges of MDA, homocysteine, and CRP were 0.39–7.06 μM (median, 1.79), 3–20 μM (median, 7.70), and 0.02–2.79 mg/dL (median, 0.39), respectively.

As a result of a comparison of biomarkers, the levels of CRP, a biomarker for oxidative stress or inflammation, and LDL-cholesterol were significantly higher in the cases than the controls, although these levels were within normal ranges (Table 2). Interestingly, dG-C8-MeIQx, a biomarker for DNA-adduct of MeIQx, was significantly higher in the cases than that of the controls, although their difference was less than 10%.

### 2.3. Epigenetic and Genetic Alterations

**Figure 1:**
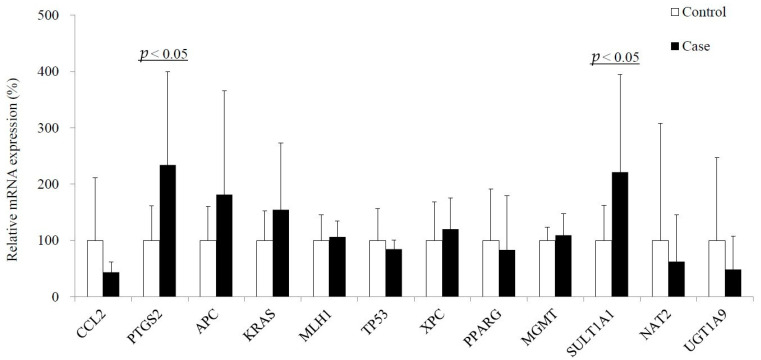
Comparison of expression of CRC-related genes: All data were normalized by reference gene *RPLP0*.

Global DNA methylation levels were measured as 5-methylcytosine (*5mC*) were somewhat higher in the cases than the controls (Table 2); however, the difference was not statistically significant. In addition, the cases showed significantly upregulated expression of *PTGS2* (*COX2*) and *SULT1A1* compared to the controls. Somewhat higher levels of *APC, KRAS,* and *XPC* were detected in the cases than the others, although they were not significant (Figure 1). The variation of each gene expression is within the possible range of the values presented in a previous report [29].

### 2.4. Alterations of Urinary Fatty Acid

Over half of the measured 31 urinary fatty acids, including linoleic acid and γ- linolenic acid, which are omega-6 series and precursors of arachidonic acid (AA), were higher in the cases than the controls (Table 3). In detail, the cases had higher levels of a total of 31 fatty acids, and categorized fatty acids including saturated fatty acids (SFA), monounsaturated fatty acids (MUFA), and polyunsaturated fatty acids (PUFA), than the controls.

### 2.5. Associations among CRC, Diet and Biomarkers

We observed positive associations between consumption of red and processed meat, which were calculated from FFQ, and levels of urinary HCAs, i.e., MeIQx and PhIP, which were quantified with LC/MS/MS (Table S2). In addition, there were positive associations between levels of urinary MeIQx or PhIP and consumption of red meat (Table S2).

For gene expression in blood, there were inverse correlations between red meat consumption and the expression of *TP53* in all subjects (Table S2). Furthermore, urinary HCA levels were positively associated with the expression of *SULT1A1*, *KRAS*, and *PTGS2*, while there was an inverse correlation between the levels of dG-C8 MeIQx and expression of *XPC*, a DNA damage repair gene (Table S2).

**Figure 2:**
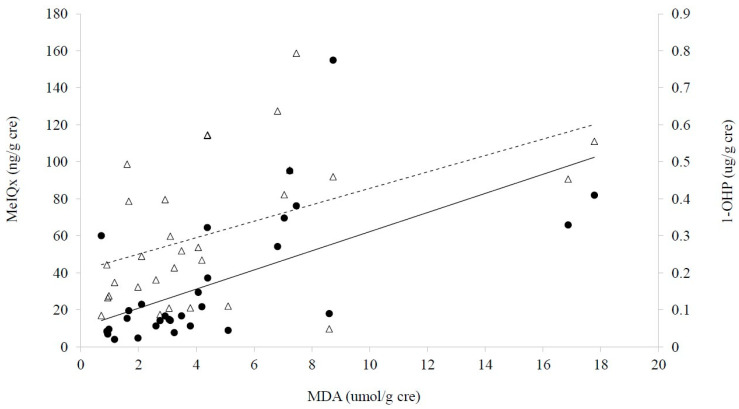
Associations among urinary MeIQx MDA, and 1 OHP levels Regression between urinary MDA and.MeIQx (0 67 P 0 01 dark circle and solid line) Regression between urinary MDA and 1 OHP ( 0 48P 0 01 triangle and dashed line) Regression between urinary MeIQx and 1 OHP ρ = 0 61 P 0 01.

Concerning exposure biomarkers, we found strong positive correlations among urinary levels of MeIQx, MDA, and 1-OHP (Figure 2). The levels of urinary 1-OHP were also positively associated with those of dG-C8 MeIQx (Table S2). Therefore, these results reflect simultaneous exposure to PAHs and HCAs, which may induce oxidative stress, which was expressed with MDA. On the other hand, tobacco smoking and fish intake were not associated with these exposure biomarkers or urinary ω-3 fatty acids (Table S3).

Urinary levels of some fatty acids, such as α-linolenic *acid* (18:3, n-3; median, 0.65 μg/L) and docosapentaenoic acid (C22:5, n-3; median, 0.18 μg/L), were positively associated with their consumption levels (medians, 0.32 g/day and 0.08 mg/day, respectively) from FFQ (*p* < 0.05). In addition, urinary levels of 10 of the 31 urinary fatty acids were positively related to the levels of red or processed meat-exposure biomarkers, i.e., urinary 1-OHP, PhIP, and MeIQx (Table 4). As half of these exposure-related fatty acids were 22 carbon chain fatty acids, we compared strength of relationship between urinary HCA levels and C22 fatty acids by the number of double bonds, 0–6. As a result, we found that these relations were strengthened by the number of double bonds (from r = 0.512 to r = 0.741 for MeIQx; from r = 0.506 to r = 0.750 for PhIP). Thus, instability of PUFA can be affected by exposure to red or processed meat.

## 3. Discussion

### 3.1. Meat Consumption and CRC in Koreans

The International Agency for research on cancer (IARC) Declaration (2015) and some reports [30] have proposed red or processed meat consumption as one of the main causes of CRC. In fact, meat consumption has jumped four-fold in Korea over the last three decades [31]. The levels of meat consumption in the present study were somewhat lower (Table 1) than the average in Korea, with 61.5 g/day for red meat and 6.0 g/day for processed meat [32], lower than the high risk amounts reported by the IARC, i.e., 100 g/day and 50 g/day, respectively, and less than one-half to two-thirds of the levels of most Western countries [1]. The Korea National Health and Nutrition Examination Survey (KNHANES), 2013–2015, showed that young people consumed more red or processed meat than the older population [33]. Our present study confirmed that age was inversely associated with processed meat consumption, regardless of CRC presence (*p* = 0.04; Table S3). In addition, the controls, who were somewhat younger than CRC patients, showed higher consumption of processed meat (Table 1). Therefore, currently, CRC in Korea may not be related to red or processed meat consumption. Although the risks of red or processed meat on CRC [1] have not yet been clearly identified in the Asian population [15], the higher consumption of processed meat by young people compared to the older population may indicate a potential risk for a future increase of CRC in Korea.

### 3.2. Exposure Levels

The average level of 1-OHP, an exposure biomarker for PAHs, was 0.13 μg/L (0.31 μg/g cre) in the present study, similar to current Korean adult levels, i.e., 0.16 μg/L, which were investigated in Korean National Environmental Health Survey (KoNEHS), 2016–2017 (adults, 19 years and older; *n* = 3787) [34]. Therefore, the present subjects may reflect the average exposure to PAHs in Koreans. For HCAs, the levels of urinary PhIP, the most prominent of the red/processed meat-induced HCAs, were considerably lower in the present study (median, 3.44 ng/L; age, 59.73 ± 12.25 years) than that in our previous study in young people (approximate average, 400 ng/L; age, 27.2 ± 7.7 years) [35]. A USA population study showed PhIP could be measured in 10% of urine samples among smokers, within a range of approx. 3–18 ng/L [36] with relatively lower sensitivity (LOQ, 4.3 ng/L) than ours (LOQ, 0.13 ng/L). In addition, a current simulation of the United States showed that the sum of PhIP and MeIQx reached to 565.3 ng/day, including 473.6 ng/day of PhIP and 91.8 ng/day of MeIQx [37]. Due to the limited degree of biomonitoring of PhIP in other countries, we compared our results to those from a study performed in Los Angeles [38,39] (African-American, 3.36; Asian-American, 3.33; Caucasian, 1.18 ng/g creatinine), and found that the levels were similar. Considering that the mean consumption of processed meat among US adults has remained unchanged in the past 18 years [40], we can estimate that the present levels of Korean urinary PhIP may be similar to current Americans levels. That is, biomonitoring results show that Koreans are exposed to similar amounts of PAHs or HCAs as Americans. Therefore, other factors, such as cooking techniques or host susceptibility to increased CRC carcinogens, can be considered as risk factors for CRC in Korea in addition to meat consumption.

### 3.3. Multiple Evidences for Effects of Red or Processed Meat on CRC

There was no direct association between CRC risk and consumption of red or processed meat in the present study. Furthermore, the controls showed higher levels of processed meat in controls than the CRC. However, we observed strong evidence that showed indirect carcinogenic mechanisms via lipid alteration, i.e., a potential series of progress for CRC: exposure to red or processed meat; alteration of metabolites, particularly fatty acids; relevant gene expression; and the onset of CRC. At first, urinary MeIQx and PhIP well reflected the intake of red meat and were positively associated with urinary 1-OHP (Table S4, Figure 2), which is a major metabolite of PAHs and reflects diet-born PAHs [35].

Secondly, red or processed meat consumption was positively associated with the amount of animal lipid or total lipid (Table S2). Sixteen individual fatty acids and all 31 fatty acids were significantly higher in the cases than the controls (Table 3). In particular, urinary levels of SFAs such as stearic acid and palmitic acid were higher in the cases than controls (Table 3). This trend supports other European results that showed the SFA stearic acid was associated with increased CRC risk [41]. Moreover, response biomarkers, e.g., MDA for lipid oxidation and multiple fatty acids for lipid metabolic alteration, were related to the exposure biomarkers for red or processed meat (Figure 2, Table 4). In particular, multiple very long-chain fatty acids (VLCFA) with 22 or more carbons showed positive correlation with the exposure biomarkers (Table 4). As fatty acid biosynthesis can be activated in diseases such as cancer, inflammation, etc. [42], VLCFA can be accumulated under such diseases and may be sensitive to CRC initiation or progress.

Thirdly, the expression of *PTGS* (*COX*)*2* in blood was significantly higher in patients than in controls (Figure 1). The levels of AA, the substrate of *PTGS2,* and α-linolenic acid, an inhibitor of *PTGS2* [43], were associated with red meat intake and with the three exposure biomarkers (Table 4). In addition, the CRC patients showed upregulation of *PTGS2*-related pro-inflammatory fatty acids, γ- linolenic acid, and eicosatrienoic acid, and compensational fatty acids, α-linolenic acid and eicosapentaenoic acid (Table 3). Moreover, the expression of *SULT1A1,* which is involved in CRC initiation via activation of HCA [44], was also related to red meat intake (Table S3), and was higher in the cases than in the controls (Table 3). Thus, the relevant gene expression also supports the association between red meat and CRC via lipid alteration.

To overcome the limitation of the small numbers of subjects in our case-control study, we did our best with systemic approaches including biological monitoring with diverse biomarkers and proper statistical approaches. Considering food intervention or lifestyle changes in CRC patients, we asked about consumption habits of red or processed meat in the previous year for the cases to avoid diet intervention for CRC. Thus, we also tried to investigate longitudinal exposure for red or processed meat consumption with HCA-DNA adducts, and found higher levels of dG-C8-MeIQx in cases than in controls (Table 2). However, the amount of consumed red or processed meat was not found to be a marker of CRC in the present study. In addition, the quantities reported by the present subjects were lower than the amounts described as “high risk” in the IARC report [1]. Moreover, controversy on the correlation between red and processed meat consumption and colorectal cancer risk was also reported in an Asian study [15]. Thus, our study suggests red or processed meat-derivative complex risks in Korean unique diet culture, such as heavy company dinner with alcohol and meat, preference of well-done steak, etc., which are related to alteration of normal lipid metabolism. 

The present results provide various biomarkers including red or processed meat-related metabolites which may be useful in the design of future studies. The present results should be confirmed by larger future studies to avoid chance errors.

## 4. Materials and Methods

### 4.1. Materials

β-Glucuronidase Type H-2 from *Helix pomatia* was purchased from Sigma-Aldrich (St. Louis, MO, USA). Internal standards (IS), such as N-(Deoxyguanosin-8-yl)-2-amino-3,8-dimethylimidazo[4,5-f] quinoxaline (dG-C8 MeIQx)-d_3_, N-(Deoxyguanosin-8-yl)-2-amino-1-methyl-6-phenylimidazo[4,5-β] pyridine (dG-C8 PhIP)-d_3_, MeIQx-d_3_, and PhIP-d_3_ and standard chemicals, MeIQx, PhIP, dG-C8 MeIQx, and dG-C8 PhIP, were purchased from Toronto Research Chemical (Toronto, Canada). Deuterated fatty acid standards, i.e., decanoic acid-d_19_, hexadecanoic acid-d_4_, oleic acid-d_17_, linoleic acid-d_11_, arachidonic acid-d_8_, eicosanoic acid-d_3_, eicosapentaenoic acid-d_5_, docosanoic acid-d_43_, and docosahexaenoic acid-d_5_, were purchased from NU-CHECK PREP Inc. (Elysian, MN, USA). All organic solvents were HPLC-or MS- (absolute) grade from Tedia (Fairfield, OH, USA). Most of the other analytical grade chemicals were obtained from Sigma-Aldrich.

### 4.2. Subjects

We recruited 15 cases and 15 controls for a case-control study on a first-come-first-served basis. The cases were newly-diagnosed with CRC at Seoul St. Mary’s Hospital, the Catholic University of Korea, Seoul, South Korea. Written informed consent was obtained from each patient using consent forms and a protocol approved by the Institutional Review Board of Seoul St. Mary’s Hospital (IRB#, KC18QNSI0057).

### 4.3. Collection of Data and Biospecimens

All subjects were interviewed and filled out a FFQ, which was developed by the National Cancer Center Korea to be specific to the consumption of red or processed meat and positively evaluated for validity and reliability for Korean diet and cancer research [45]. The cases were asked about food consumption in their daily lives for a year before CRC-diagnoses to avoid recent food intervention due to the diagnosis or heath status, while the controls were asked about their current consumption habits. After obtaining informed consent, we collected 40 mL of urine and 13 mL of peripheral blood divided into three different tubes: 5 mL in an EDTA tube, 5 mL in a clot activator gel tube, and 3 mL in a DNA/RNA Shield™ (Zymo Research, Irvine, CA, USA). The EDTA blood tube samples were centrifuged at 14,000 rpm at 4 °C, and buffy coats and plasma fraction were separated. These fractions were stored at −20 °C before experiments. The peripheral blood in the clot activator gel tube was centrifuged as described for EDTA tubes and the separated serum fraction was used to analyze hematological indicators, such as aspartate transaminase (AST), alanine aminotransferase (ALT), C-reactive protein (CRP), total cholesterol (TC), triglyceride (TG), low density lipoprotein (LDL-cholesterol), high density lipoprotein (HDL-cholesterol), and homocysteine with an automatic biomedical analyzer (HITACHI 7020, Tokyo, Japan).

### 4.4. Analyses of Urinary 1-OHP

Urinary 1-OHP was measured for red or processed meat-induced PAHs, as described in our previous study [35]. In brief, 200 μL of urine was mixed with 200 μL of 0.2M sodium acetate buffer (pH 5.0) and 30 μL of β-glucuronidase (2550 units; Sigma-Aldrich). After 5 h of incubation at 37 °C, 570 μL of acetonitrile (ACN) was added to the mixture. After centrifuging at 14,000 rpm for 10 min, 400 μL of the supernatant was transferred to an HPLC vial and 100 μL of the vial liquid was injected into an HPLC/FLD system, which consisted of a YL9111 binary pump (Yonglin Co., Seoul, Korea), a YL9150 autosampler (Yonglin Co.), a Jasco FP-2020 plus FD (Jasco, Tokyo, Japan), and a YMC-Triart C18 column (150 mm × 4.6 mm, 3.0 um; YMC Co. Ltd., Kyoto, Japan). The mobile phase was 65% ACN in water. Excitation and emission wavelengths were 242 and 388 nm, respectively.

### 4.5. Analyses of Urinary HCAs and of HCA-DNA Adducts

We analyzed MeIQx and PhIP for exposure to red or processed meat-induced HCAs in urine. In brief, we added 4 μL of IS, i.e., 0.57 uM of MeIQx-d_3_ and 0.26 uM of PhIP-d_3_, to 2 mL of urine and hydrolyzed the mixture with 200 μL of 10 N NaOH. The mixtures were extracted twice with 6 mL of CH_2_Cl_2_ and the extracts were dried in a SpeedVac concentrator (Savant Inc., Farmingdale, NY, USA) and dissolved in 100 μL of 50% of ACN. After centrifuging, 50 μL of the supernatant was transferred to a vial of UPLC-MS/MS, which was configured with an Agilent 1290 Infinity II system (Agilent Technologies, Santa Clara, CA, USA). The column used in the UHPLC system was Poroshell 120 SB-C18 (100 × 3.0 mm, 2.7 μm). The mobile phase was a binary mixture of deionized water containing 0.01% of formic acid and 20 mM of ammonium formate (A) and ACN (B). These two mobile phases were used in a gradient mode at a flow rate of 0.4 mL/min. The gradient conditions were 5% of B for 1 min, increasing B to 95% over 8 min with a linear gradient, retaining 95% of B for 2 min to wash the column, and then switching to 5% of B for 3 min. Five μL of each sample was injected into the UHPLC system. The column temperature was maintained at 35 °C.

For MS, we used Agilent Triple Quadrupole 6495 system with a specialized type of ESI interface, Agilent Jet Stream source. The electric parameters were set up as positive mode, 4000 V capillary voltage of the electrospray ionization source, 2000 V nozzle voltage, 200 V high pressure RF voltage, 100 V low pressure RF voltage for dual ion funnel and 380 V fragment voltage. For drying liquids from UHPLC, nebulizer gas flow and sheath gas flow were 12 and 10 L/min, respectively. The temperatures of nebulizer and sheath gases were 290 °C and 250 °C, respectively.

We also analyzed two major HCA-DNA adducts, dG-C8-MeIQx and dG-C8-PhIP, from genomic DNA samples. In brief, genomic DNA was extracted from buffy coat samples of peripheral blood from the subjects, which was collected in the EDTA tube with a QIAamp DNA Blood Mini Kit (Qiagen, Hilden, Germany) according to manufacturer’s instructions. DNA concentration and purity were analyzed with NanoDrop One Microvolume UV-Vis Spectrophotometer (Thermo Fisher Scientific, Wilmington, DE, USA). All DNA samples, for which 260/280 and 260/230 ratios were greater than 1.7, were stored for further analyses. If the DNA samples did not satisfy the previous ratios, we purified them with Genomic DNA Clean & Concentrator^TM^ Kit (Zymo Research). According to the manufacturer’s protocol, we digested the same amount (1.766 ug) of DNA samples with DNA Degradase Plus^TM^ Kit (Zymo Research). Finally, we analyzed dG-C8 MeIQx and dG-C8 PhIP with the same UPLC-MS/MS system and conditions as for HCAs.

Transitions of multiple reaction monitoring (MRM) and their conditions for MeIQx, dG-C8- MeIQx, PhIP, and dG-C8- PhIP including their internal standards, MeIQx-d_3_, dG-C8- MeIQx-d_3_, PhIP-d_3_, and dG-C8- PhIP-d_3,_ are described in Table S4. For optimization and validation of analyses, data of accuracy and calibration curves are on Supplementary Materials (File S1). All the urinary analytes were adjusted for creatinine, which was calculated by our previous methods with the HPLC/UVD method [34].

### 4.6. Analyses of Urinary MDA

Urinary MDA was measured for lipid oxidation and oxidative stress as previously described [24], with some modification. Twenty-three mM of thiobarbiturate (TBA) reagent was prepared by dissolving 66.3 mg of TBA in 20 mL of water. Ten mM of MDA standard solution was prepared by adding 123.6 μL of 1,1,3,3-tetraethoxypropane in 50 mL of 40% of ethanol. Working MDA standards of 0.625, 1.25, 2.5, and 5 uM were made every day from the 10 mM MDA stock solution. The reaction mixtures consisted of 300 μL of 0.5 M of phosphoric acid, 150 μL of TBA reagent, and 50 μL of each MDA standard or urine samples. The mixtures were heated at 95 °C for 1 h, then chilled on ice for 5 min. Five hundred μL of methanol was added to the mixture. After centrifuging at 14,000 rpm for 5 min, we transferred the supernatant to an HPLC vial and injected 20 μL of the sample into the HPLC/UVD system, which was the same as the above HPLC system with a Shimadzu SPD-10A UV/VIS Detector (Shimadzu Corporation, Tokyo, Japan). The levels of TBA-MDA adducts were determined at 532 nm. The YMC-Triart C18 column was eluted with 50 mM of potassium phosphate buffer (pH 6.8) and methanol (58:42, *v/v*). Mobile phase flow rate was 0.6 mL/min.

### 4.7. Analyses of Global DNA Methylation

We analyzed global DNA methylation levels in the above genomic DNA samples with the MethylFlash Methylated DNA Quantification kit (EpigenTek, Brooklyn, NY, USA), following our previous method [34]. In brief, 100 ng of each genomic DNA was bound to strip wells. The 5-methylcytosine (5-mC) antigen-antibody-complex was analyzed at 450 nm with EL × 800 Microplate spectrophotometer (Bio-Tek, Winooski, VT, USA). The amount of 5-mC (%) was quantified from a standard curve (0.5–10.0 ng).

### 4.8. Quantification of Gene Expression

Total RNA was isolated from 3 mL of each whole blood sample in a DNA/RNA Shield^TM^ Blood Collection Tube (Zymo research) with the Quick-RNA™ Whole blood kit (Zymo research). The final RNA was eluted with 15 μL of DNase/RNase-Free water. The RNA eluate was qualitatively assessed and quantified with LabChip GX (Caliper, Hopkinton, MA, USA) and Nanodrop 2000 (Thermo science, Waltham, MA, USA). The cDNA was synthesized from 100 ng of the RNA eluate with *AccuPower*^®^ Rocket Script Cycle RT PreMix (Bioneer, Daejeon, Korea) on MyGenie 96 Gradient Thermal Block (Bioneer), according to the manufacturer’s protocol. The RT was performed under the following thermo-cycling conditions: 12 cycles at 37 °C for 30 s, 48 °C for 4 min, and 55 °C for 30 s followed by 1 cycle at 95 °C for 10 min. After the RT, the Real-time PCR array was performed on Exicycler 96 Real-Time Quantitative Thermal Block with the following cycling parameters: 40 cycles at 95 °C for 5 s, 58 °C for 25 s, and 72 °C for 30 sec. The reaction mixture for the real-time PCR was assembled with 25 μL of *AccuPower*^®^ 2X GreenStar Master Mix (Bioneer), 15 μL of deionized sterile water, 5 μL of sense and antisense primers (final concentration, 3 uM for each), and 5 μL of cDNA (final concentration, 100 ng per μL) in 50 μL of total volume.

All primers for the 15 target genes, i.e., *CCL2, PTGS2, APC, KRAS, MLH1, TP53, XPC, LEP, PPARG, APOA1, MGMT, CYP1A2, SULT1A1, NAT2, UGT1A9,* and a reference gene, *RPLP0*, were designed with the Primer3 software based on published sequences in the National Center for Biotechnology Information (NCBI) and synthesized by Bioneer. Table S2 shows the primer sequences. The expression levels of target genes were calculated as the 2^−ΔΔCt^ method and normalized against *RPLP0*. Each plate included 7 or 8 pairs of age and sex-matched controls and cases. We accepted data with CV (coefficient of variation) <10% from duplicated expression results.

### 4.9. Analyses of Urinary Fatty Acids

We analyzed 31 representative fatty acids in urine with minor modifications to previous methods [23,46]. In brief, the internal standard mixture, i.e., decanoic acid-d_19_, hexadecanoic acid-d_4_, oleic acid-d_17_, linoleic acid-d_11_, arachidonic acid-d_8_, eicosanoic acid-d_3_, eicosapentaenoic acid-d_5_, docosanoic acid-d_43_, and docosahexaenoic acid-d_5_ (100 ng/50 μL), was added to 100 μL of urine sample. Extraction was performed twice with 2 mL of chloroform and mixed with 1 mL of methanol. The mixture was hydrolyzed with 1 mL of 0.5 M HCl. The hydrolyzed fractions were converted to pentafluorobenzyl esters with a mixture of pentafluorobenzyl bromide in ACN solution (1:19 *v/v*, 25 μL) and N, N-diisopropylethylamine in ACN solution (1:9 *v/v*, 25 μL). Finally, the derivatized fatty acids were re-dissolved in 100 μL of n-dodecane and 1 μL of the derivatized extract was injected onto a 7890 A GC System coupled to a 7000 MS Triple Quad (Agilent Technologies). Each sample was analyzed on a Zebron ZB-1MS Capillary GC Column (30 m × 0.25 mm × 0.50 µm; Phenomenex Inc., Torrance, CA, USA).

### 4.10. Statistical Analysis

The Shapiro-Wilk W Test was used to test distributional normality in all biomarkers. Pearson’s product moment or Spearman’s rank correlation analyses were used to study univariate relations between each dietary factor and CRC potential biomarker. ANOVA or Kruskal-Wallis were used to find differences in biomarkers between cases and controls. Multiple regression analyses were used for multivariate relationships among biomarkers and CRC risks.

Statistical significance was considered at *p* < 0.05. The JMP ver. 4 (SAS Institute, Cary, NC, USA) was used for all statistical analyses.

## 5. Conclusions

We could not find a direct association between CRC risk and consumption of red or processed meat in the present study; however, we did observe strong evidence that showed indirect carcinogenic mechanisms via lipid alteration with a series of processes including exposure to red meat, alteration of fatty acids, and relevant gene expression.

## Figures and Tables

**Table 1:** Characteristics of all subjects.

Variables	Control (*n* = 15)		CRC (*n* = 15)		*p*-Value
	Mean	STD ^c^	Mean	STD	
Age (years)	56.40	13.12	63.07	10.74	0.14
Height (cm)	164.21	10.60	160.25	8.22	0.26
Sex (*n* of men)	8		7		0.72
Tobacco smoking (ppd) ^a^	8.00	16.67	7.67	16.13	0.56
Body mass index(kg/m^2^)	25.53	2.49	22.73	3.49	0.02 *
Energy intake (kcal/day)	1730.16	826.63	1500.30	638.85	0.43
Meat and lipid intake ^b^ (g/day)					
Red meat	57.08	39.53	50.92	33.22	0.71
Processed meat	4.79	6.13	0.31	0.87	<0.01 **
Vegetable lipid	17.02	11.64	15.63	10.07	0.73
Animal lipid	22.08	13.46	15.88	8.29	0.14
Total lipid	39.10	22.63	31.51	13.38	0.73
Frequency of fish intake (*n*)					0.47 ^d^
Rare	6		9		
a piece/2 days	9		6		
a piece/day	0		0		

^a^ Park per day, ^b^ Current consumption for controls; one year ago-consumption for CRC cases, ^c^ Standard deviation, ^d^ Fisher’s exact test; other *p* values were calculated by ANOVA or Wilcoxon Sign-Rank test due to normality, * *p* < 0.05, ** *p* < 0.01.

**Table 2:** Comparison of biomarkers between controls and CRC.

Biomarkers	Controls (*n* = 15)		CRC (*n* = 15)		*p*-Value	Adjusted *p*-Value ^a^	Normal Range
	Mean	STD	Mean	STD			
Hematological biomarkers							
AST (U)	25.53	9.11	24.2	8.28	0.79	0.48	10–40
ALT (U)	27.67	12.92	21.73	9.41	0.14	0.19	7–56
CRP (mg/dL)	0.92	0.81	0.32	0.26	0.03 *	<0.01 **	0.5–1.0
TC (mg/dL)	170	44.93	182.33	30.27	0.39	0.45	<200
TG (mg/dL)	160.6	160.54	121.33	47.76	0.92	0.62	<150
LDL-C (mg/dL)	90.11	35.19	114.67	24.54	0.04 *	0.06†	<130
HDL-C (mg/dL)	43.4	14.28	43.27	11.27	0.98	0.82	≥40
Homocysteine (μM)	6.87	2.13	8.8	3.93	0.09	0.10	<15
Exposure or response biomarkers							
MDA (μM)	2.23	2.13	2.18	1.21	0.28	0.65	
1-OHP (μg/L)	0.12	0.04	0.16	0.06	0.06	0.09 ^†^	
MeIQx (ng/L)	17.07	23.19	12.21	5.91	0.44	0.80	
PhIP (ng/L)	7.84	2.65	11.96	2.65	0.28	0.44	
dG-C8 MeIQx/1.766ug of DNA	5.17	0.07	5.29	0.11	<0.01 **	<0.01 **	
Global DNA methylation (%)	4.96	1.39	5.64	1.00	0.17	0.15	

^a^ Adjusted for age and sex; ^†^ borderline significance (0.05 < *p* < 0.1), * *p* < 0.05, ** *p* < 0.01.

**Table 3:** Comparison of 32 fatty acid levels between controls and CRC.

Contents		Control (*n* = 15)		CRC (*n* = 15)		*p*-Value ^a^
		Mean (ng/mL)	STD	Mean (ng/mL)	STD	
C14:0	Myristic acid	73.12	50.25	147.04	129.71	0.04 *
C14:1	Myristoleic acid	4.67	4.4	7.08	4.61	0.11
C15:0	Pentadecanoic acid	14.41	6.3	30.21	25.04	0.02 *
C15:1	Pentadecenoic acid	0.58	0.73	0.7	0.45	0.10
C16:0	Palmitic acid	452.35	359.51	843.96	659.54	0.03 *
C16:1	Palmitoleic acid	11.03	15.36	28.7	26.43	0.01 *
C16:1T	Palmitelaidic acid	2.73	3.81	7.12	6.56	0.01 *
C17:0	Heptadecanoic acid	12.66	12.92	51.69	76.51	0.03 *
C18:0	Stearic acid	384.38	233.25	819.2	671.79	0.03 *
C18:1 Mix	Vaccenic acid, Oleic acid, Elaidic acid	147.57	203.45	411.62	306.6	<0.01 **
C18:2 Mix	Linoleic acid, Linolelaidic acid	23.41	26.84	54.38	30.27	<0.01 **
C18:3	α-Linolenic acid	0.84	1.01	0.95	0.56	0.04 *
C18:3	γ- Linolenic acid	26.19	27.54	58.21	25.79	<0.001 ***
C19:0	Nonadecylic acid	1.05	0.6	5.34	7.28	<0.01 **
C19:1	7- Nonadecylic acid	0.88	0.2	1.79	0.2	<0.01 **
C20:0	Arachidic acid	5.03	2.15	20.34	24.01	<0.01 **
C20:1 Mix	11- Eicosenoic acid	3.3	3.92	6.47	7.25	0.06
C20:2	11-14- Eicosadienoic acid	1.1	0.87	1.36	1.33	0.60
C20:3	11-14-17 Eicosatrienoic acid	1.07	1.47	2.44	4.33	0.09
C20:3	Homogamma linolenic acid	3.33	3.88	8.2	6.26	<0.01 **
C20:4	Arachidonic acid	11.37	2.23	14.95	2.23	0.27
C20:5	Eicosapentaenoic acid	4.89	5.33	11.9	9.23	<0.01 **
C22:0	Behenic acid	8.91	3.52	15.93	11.2	0.07
C22:1	Erucic acid	3.28	1.17	3.02	2.11	0.08
C22:2	Docosadienoic acid	0.47	0.45	0.51	0.36	0.47
C22:3	Docosatrienoic acid	0.19	0.23	0.22	0.17	0.09
C22:4	Docosatetraenoic acid	0.15	0.09	0.2	0.12	0.10
C22:5	ω-3 Docosapentaenoic acid	0.22	0.2	0.3	0.18	0.13
C22:6	Docosahexaenoic acid	2.43	1.42	3.11	1.54	0.14
C24:0	Lignoceric acid	4.34	1.32	7.85	7.8	0.05
C24:1	Nervonic acid	1.29	0.68	1.54	1	0.60
Total		1207.26	848.67	2566.32	1883.83	<0.01 **
SAFA		956.25	653.39	1941.54	1533.67	0.01 *
MUFA		175.34	230.12	468.04	346.87	<0.01 **
PUFA		75.66	72.43	156.74	68.06	<0.01 **

^a^ Mann-Whitney u-test or Student’s *t*-test; * *p* < 0.05, ** *p* < 0.01, *** *p* < 0.001

**Table 4:** Exposure biomarkers-associated fatty acids.

Fatty Acids	by Variable	Correlation (r)	*p*-Value
C18:0 Stearic acid (μg/g cre)	1-OHP (μg/g cre)	0.563	0.001 **
	MeIQx (ng/g cre)	0.391	0.033 *
	PhIP (ng/g cre)	0.429	0.018 *
C18:3 α-Linolenic acid (μg/g cre)	1-OHP (μg/g Cre)	0.373	0.042 *
	MeIQx (ng/g cre)	0.372	0.043 *
	PhIP (ng/g cre)	0.386	0.035 *
C20:4 Arachidonic acid (μg/g cre)	1-OHP (μg/g Cre)	0.491	0.006 **
	MeIQx (ng/g cre)	0.537	0.002 **
	PhIP (ng/g cre)	0.585	<0.001 ***
C22:0 Behenic acid (μg/g cre)	1-OHP (μg/g Cre)	0.575	<0.001 ***
	MeIQx (ng/g cre)	0.512	0.004 **
	PhIP (ng/g cre)	0.506	0.004 **
C22:1 Erucic acid (μg/g cre)	1-OHP (μg/g Cre)	0.742	<0.001 ***
	MeIQx (ng/g cre)	0.646	<0.001 ***
	PhIP (ng/g cre)	0.446	0.013 *
C22:4 Docosatetraenoic acid (μg/g cre)	1-OHP (μg/g Cre)	0.537	0.002 **
	MeIQx (ng/g cre)	0.572	<0.001 ***
	PhIP (ng/g cre)	0.570	<0.001 ***
C22:5 ω-3 Docosapentaenoic acid (μg/g cre)	1-OHP (μg/g Cre)	0.492	0.006 **
	MeIQx (ng/g cre)	0.584	<0.001 ***
	PhIP (ng/g cre)	0.629	<0.001 ***
C22:6 Docosahexaenoic acid (μg/g cre)	1-OHP (μg/g Cre)	0.579	<0.001 ***
	MeIQx (ng/g cre)	0.741	<0.001 ***
	PhIP (ng/g cre)	0.750	<0.001 ***
C24:0 Lignoceric acid (μg/g cre)	1-OHP (μg/g Cre)	0.431	0.017 *
	MeIQx (ng/g cre)	0.462	0.010 *
	PhIP (ng/g cre)	0.436	0.016 *
C24:1 Nervonic acid (μg/g cre)	1-OHP (μg/g Cre)	0.566	0.001 **
	MeIQx (ng/g cre)	0.607	<0.001 **
	PhIP (ng/g cre)	0.595	<0.001 ***

* *p* < 0.05, ** *p* < 0.01, *** *p* < 0.001.

## Data Availability

Data is contained within the article and supplementary material.

## Supplementary Materials

The following are available online at https://www.mdpi.com/article/10.3390/metabo11070462/s1, Table S1: Characteristics of CRC cases, Table S2: Significant associations among CRC, diet and biomarkers, Table S3: MRM channels for 8 compounds including internal standards of HCAs and dG-HCAs, Table S4: Sequences of the sense and antisense primers used for quantitative real time PCR, File S1: Calibration curves for HCAs.

## Abbreviations

2-Amino-3:8-dimethylimidazo[4,5-f] quinoxaline (MeIQx), 2-Amino-1-methyl-6-phenylimidazo[4,5-b]pyridine (PhIP), alanine amino transferase (ALT), aspartate amino transferase (AST), low density lipoprotein cholesterol (LDL-C), colorectal cancer (CRC), C-reactive protein (CRP), deoxyguanosine (dG), food frequency questionnaire (FFQ), heterocyclic amines (HCAs), high density lipoprotein cholesterol (HDL-C), hydroxoxypyrene (1-OHP), International Agency for research on cancer (IARC), (limit of quantification (LOQ), malondialdehyde (MDA), polycyclic aromatic hydrocarbons (PAHs), saturated fatty acids (SFAs), total cholesterol (*TC*), triglyceride (*TG*), the World Health Organization (WHO), very long chain fatty acids (VLCFA).
