# Supplementary material for: Metabolic Evidence Rather Than Amounts of Red or Processed Meat as a Risk on Korean Colorectal Cancer
Source: Metabolites. 2021 Jul 16;11(7):462. doi: 10.3390/metabo11070462 (PMC8303103; doi:10.3390/metabo11070462)
Supplement: Supplementary file 1 [file metabolites-11-00462-s001.zip › metabolites-1283725-supplementary.pdf]

Table S1. Characteristics of CRC cases

ID	location	stage
CMC1-01	rectum	1
CMC1-02	rectum	2
CMC1-03	descending colon	3
CMC1-04	rectum	3
CMC1-05	sigmoid colon	2
CMC1-06	descending colon	3
CMC1-07	rectosigmoid colon	2
CMC1-08	ascending colon	1
CMC1-09	descending colon	3
CMC1-10	rectum	1
CMC1-11	rectosigmoid colon	4
CMC1-12	ascending colon	3
CMC1-13	sigmoid colon	1
CMC1-15	rectosigmoid colon	3
CMC1-16	rectum	3

Table S2. Significant associations among CRC, diet and biomarkers

Variable	by Variable	ρ	*P*-value
1-OHP (ug/L)	C22:1/cre (ug/L)	-0.37	0.04
1-OHP (ug/L)	C22:4/cre (ug/g cre)	-0.35	0.06
1-OHP (ug/L)	C22:6/cre (ug/g cre)	-0.40	0.03
1-OHP (ug/L)	C24:1/cre (ug/g cre)	-0.37	0.05
1-OHP with Cre (ug/g Cre)	C14:0/cre (ug/g cre)	0.57	0.00
1-OHP with Cre (ug/g Cre)	C14:1/cre (ug/g cre)	0.38	0.04
1-OHP with Cre (ug/g Cre)	C15:0/cre (ug/g cre)	0.60	0.00
1-OHP with Cre (ug/g Cre)	C15:1 (ug/L)	-0.50	0.01
1-OHP with Cre (ug/g Cre)	C16:0/cre (ug/g cre)	0.60	0.00
1-OHP with Cre (ug/g Cre)	C16:1/cre (ug/g cre)	0.36	0.05
1-OHP with Cre (ug/g Cre)	C16:1T/cre (ug/g cre)	0.36	0.05
1-OHP with Cre (ug/g Cre)	C17:0/cre (ug/g cre)	0.33	0.07
1-OHP with Cre (ug/g Cre)	C18:0/cre (ug/g cre)	0.56	0.00
1-OHP with Cre (ug/g Cre)	C18:1 Mix/cre (ug/g cre)	0.37	0.05
1-OHP with Cre (ug/g Cre)	C18:2 Mix/cre (ug/g cre)	0.39	0.03
1-OHP with Cre (ug/g Cre)	C18:3 Alpha Linolenate/cre (ug/g cre)	0.37	0.04
1-OHP with Cre (ug/g Cre)	C18:3 Gamma Linolenate/cre (ug/g cre)	0.44	0.02
1-OHP with Cre (ug/g Cre)	C19:1/cre (ug/g cre)	0.63	0.00
1-OHP with Cre (ug/g Cre)	C20:1 Mix/cre (ug/g cre)	0.42	0.02
1-OHP with Cre (ug/g Cre)	C20:2/cre (ug/g cre)	0.54	0.00
1-OHP with Cre (ug/g Cre)	C20:4 (ug/L)	-0.63	0.00
1-OHP with Cre (ug/g Cre)	C20:4/cre (ug/g cre)	0.49	0.01
1-OHP with Cre (ug/g Cre)	C22:0/cre (ug/g cre)	0.57	0.00
1-OHP with Cre (ug/g Cre)	C22:1 (ug/L)	0.40	0.03
1-OHP with Cre (ug/g Cre)	C22:1/cre (ug/g cre)	0.74	0.00
1-OHP with Cre (ug/g Cre)	C22:2/cre (ug/g cre)	0.55	0.00
1-OHP with Cre (ug/g Cre)	C22:3/cre (ug/g cre)	0.49	0.01
1-OHP with Cre (ug/g Cre)	C22:4 (ug/L)	-0.46	0.01
1-OHP with Cre (ug/g Cre)	C22:4/cre (ug/g cre)	0.54	0.00
1-OHP with Cre (ug/g Cre)	C22:5 (ug/L)	-0.51	0.00
1-OHP with Cre (ug/g Cre)	C22:5/cre (ug/g cre)	0.49	0.01
1-OHP with Cre (ug/g Cre)	C22:6 (ug/L)	-0.63	0.00
1-OHP with Cre (ug/g Cre)	C22:6/cre (ug/g cre)	0.58	0.00
1-OHP with Cre (ug/g Cre)	C24:0/cre (ug/g cre)	0.43	0.02
1-OHP with Cre (ug/g Cre)	C24:1 (ug/L)	-0.46	0.01
1-OHP with Cre (ug/g Cre)	C24:1/cre (ug/g cre)	0.57	0.00
1-OHP with Cre (ug/g Cre)	CCL2	-0.62	0.08

1-OHP with Cre (ug/g Cre)	MeIQx with Cre (ng/g cre)	0.61	0.00
1-OHP with Cre (ug/g Cre)	MUFA/cre (ug/g cre)	0.38	0.04
1-OHP with Cre (ug/g Cre)	PhIP with Cre (ng/g cre)	0.52	0.00
1-OHP with Cre (ug/g Cre)	PUFA/cre (ug/g cre)	0.45	0.01
1-OHP with Cre (ug/g Cre)	SAFA/cre	0.60	0.00
1-OHP with Cre (ug/g Cre)	total fatty acid/cre	0.57	0.00
1-OHP with Cre (ug/g Cre)	TP53	-0.39	0.07
1-OHP with Cre (ug/g Cre)	UFA/cre (ug/g cre)	0.40	0.03
5-mC (ng=%)	C15:1/cre (ug/g cre)	-0.40	0.03
5-mC (ng=%)	C18:3 Alpha Linolenate (ug/L)	-0.37	0.04
5-mC (ng=%)	C24:0 (ug/L)	0.33	0.08
Age (years)	BW (kg)	-0.46	0.01
Age (years)	Height (cm)	-0.45	0.01
Age (years)	MeIQx (ng/L)	-0.34	0.06
Age (years)	MLH1	0.47	0.02
Age (years)	Processed meat (g)	-0.39	0.04
Age (years)	XPC	0.37	0.09
Age (years)	Total lipid (g)	-0.35	0.06
ALT (U)	C20:5 (ug/L)	-0.32	0.08
ALT (U)	C22:2 (ug/L)	-0.34	0.06
ALT (U)	CRP (mg/dL)	0.41	0.03
ALT (U)	Fiber (g)	0.39	0.04
ALT (U)	Folate (*μ*g)	0.36	0.05
ALT (U)	LDLC (mg/dL)	-0.44	0.01
APC	KRAS	0.91	0.00
APC	MGMT	-0.51	0.01
APC	SULT1A1	0.83	0.00
AST (U)	ALT (U)	0.69	0.00
AST (U)	C22:4/cre (ug/g cre)	0.40	0.03
AST (U)	C22:6/cre (ug/g cre)	0.31	0.09
AST (U)	C24:1/cre (ug/g cre)	0.34	0.06
AST (U)	CCL2	-0.62	0.08
AST (U)	Fiber (g)	0.33	0.08
AST (U)	LDLC (mg/dL)	-0.37	0.05
AST (U)	MDA (umol/L)	-0.37	0.05
AST (U)	UGT1A9	0.61	0.02
BMI (kg/m^2)	1-OHP (ug/L)	-0.32	0.08
BMI (kg/m^2)	CRP (mg/dL)	0.36	0.05
BMI (kg/m^2)	dG-C8 MeIQx/1.766ug of DNA	-0.32	0.09
BMI (kg/m^2)	MLH1	0.49	0.02
BMI (kg/m^2)	Processed meat (g)	0.39	0.04

BW (kg)	1-OHP (ug/L)	-0.42	0.02
BW (kg)	BMI (kg/m^2)	0.77	0.00
BW (kg)	C18:2 Mix/cre (ug/g cre)	-0.35	0.06
BW (kg)	C18:3 Gamma Linolenate/cre (ug/g cre)	-0.39	0.03
BW (kg)	PPARG	0.45	0.05
BW (kg)	Processed meat (g)	0.41	0.03
BW (kg)	PUFA/cre (ug/g cre)	-0.37	0.04
BW (kg)	SULT1A1	-0.36	0.09
CCL2	C22:5 (ug/L)	0.73	0.03
CCL2	NAT2	1.00	0.00
CCL2	TP53	0.92	0.00
CCL2	UGT1A9	0.94	0.00
CCL2	XPC	-0.84	0.01
CRP (mg/dL)	C20:4 (ug/L)	-0.34	0.06
CRP (mg/dL)	C22:2/cre (ug/g cre)	0.32	0.08
CRP (mg/dL)	C22:3/cre (ug/g cre)	0.35	0.05
dG-C8 MeIQx/1.766ug of DNA	1-OHP (ug/L)	0.31	0.09
dG-C8 MeIQx/1.766ug of DNA	C18:2 Mix (ug/L)	0.34	0.07
dG-C8 MeIQx/1.766ug of DNA	C18:3 Gamma Linolenate (ug/L)	0.35	0.06
dG-C8 MeIQx/1.766ug of DNA	C19:1 (ug/L)	0.35	0.06
dG-C8 MeIQx/1.766ug of DNA	C20:1 Mix (ug/L)	0.32	0.08
dG-C8 MeIQx/1.766ug of DNA	C20:2 (ug/L)	0.33	0.08
dG-C8 MeIQx/1.766ug of DNA	C20:3 Eicosatrienoate (ug/L)	0.31	0.09
dG-C8 MeIQx/1.766ug of DNA	C20:3 Homogamma Linolenate (ug/L)	0.52	0.00
dG-C8 MeIQx/1.766ug of DNA	C20:5 (ug/L)	0.53	0.00
dG-C8 MeIQx/1.766ug of DNA	C20:5/cre (ug/g cre)	0.32	0.09
dG-C8 MeIQx/1.766ug of DNA	C22:6/cre (ug/g cre)	-0.34	0.07
dG-C8 MeIQx/1.766ug of DNA	C24:0 (ug/L)	0.32	0.09
dG-C8 MeIQx/1.766ug of DNA	MDA with Cre (uM/g cre)	-0.46	0.01
dG-C8 MeIQx/1.766ug of DNA	MeIQx with Cre (ng/g cre)	-0.43	0.02
dG-C8 MeIQx/1.766ug of DNA	PhIP with Cre (ng/g cre)	-0.39	0.03
dG-C8 MeIQx/1.766ug of DNA	PUFA	0.41	0.02
dG-C8 MeIQx/1.766ug of DNA	XPC	-0.47	0.03
Fiber (g)	C15:1 (ug/L)	0.64	0.00
Fiber (g)	C15:1/cre (ug/g cre)	0.52	0.00
Fiber (g)	Folate (μg)	0.97	0.00
Fiber (g)	Fruit (g)	0.57	0.00
Fiber (g)	Kimchi (g)	0.78	0.00
Fiber (g)	UGT1A9	0.70	0.01
Fiber (g)	Vegetable lipid (g)	0.65	0.00

Fiber (g)	Total lipid (g)	0.44	0.02
Folate (μg)	C15:1 (ug/L)	0.59	0.00
Folate (μg)	C15:1/cre (ug/g cre)	0.50	0.01
Folate (μg)	Fruit (g)	0.61	0.00
Folate (μg)	Kimchi (g)	0.76	0.00
Folate (μg)	PhIP (ng/L)	-0.32	0.09
Folate (μg)	PPARG	-0.44	0.06
Folate (μg)	UGT1A9	0.60	0.02
Folate (μg)	Vegetable lipid (g)	0.61	0.00
Folate (μg)	Total lipid (g)	0.43	0.02
Fruit (g)	C15:1 (ug/L)	0.37	0.05
Fruit (g)	C22:5 (ug/L)	0.46	0.01
Fruit (g)	CCL2	0.82	0.01
Fruit (g)	Kimchi (g)	0.39	0.04
Fruit (g)	MDA (umol/L)	0.38	0.04
Fruit (g)	NAT2	0.79	0.00
Fruit (g)	TP53	0.40	0.07
Fruit (g)	UGT1A9	0.49	0.08
HDLC (mg/dL)	C14:1/cre (ug/g cre)	0.35	0.06
HDLC (mg/dL)	C18:2 Mix/cre (ug/g cre)	0.34	0.06
HDLC (mg/dL)	C18:3 Alpha Linolenate/cre (ug/g cre)	0.34	0.06
HDLC (mg/dL)	C18:3 Gamma Linolenate/cre (ug/g cre)	0.45	0.01
HDLC (mg/dL)	C20:5/cre (ug/g cre)	0.34	0.06
HDLC (mg/dL)	C22:2/cre (ug/g cre)	0.37	0.04
HDLC (mg/dL)	C22:4 (ug/L)	-0.36	0.05
HDLC (mg/dL)	C24:1 (ug/L)	-0.39	0.03
HDLC (mg/dL)	C24:1/cre (ug/g cre)	-0.40	0.03
HDLC (mg/dL)	PPARG	-0.46	0.04
HDLC (mg/dL)	PUFA/cre (ug/g cre)	0.37	0.04
HDLC (mg/dL)	UFA/cre (ug/g cre)	0.32	0.09
Height (cm)	AST (U)	-0.32	0.09
Height (cm)	BW (kg)	0.64	0.00
Height (cm)	C15:1/cre (ug/g cre)	-0.36	0.05
Height (cm)	C18:3 Gamma Linolenate/cre (ug/g cre)	-0.34	0.06
Height (cm)	C20:5/cre (ug/g cre)	-0.33	0.07
Height (cm)	Fiber (g)	-0.32	0.09
Height (cm)	Folate (μg)	-0.35	0.06
Height (cm)	HDLC (mg/dL)	-0.38	0.04

Height (cm)	MeIQx (ng/L)	0.31	0.09
Height (cm)	PPARG	0.41	0.08
Height (cm)	PUFA/cre (ug/g cre)	-0.32	0.09
Height (cm)	TG (mg/dL)	0.31	0.09
Height (cm)	UGT1A9	-0.52	0.06
Homocystein (uM)	C14:1/cre (ug/g cre)	0.34	0.07
Homocystein (uM)	C15:0 (ug/L)	0.37	0.04
Homocystein (uM)	C15:0/cre (ug/g cre)	0.33	0.07
Homocystein (uM)	C16:0 (ug/L)	0.41	0.03
Homocystein (uM)	C16:0/cre (ug/g cre)	0.33	0.07
Homocystein (uM)	C19:1/cre (ug/g cre)	0.31	0.09
Homocystein (uM)	C20:1 Mix (ug/L)	0.50	0.00
Homocystein (uM)	C20:1 Mix/cre (ug/g cre)	0.48	0.01
Homocystein (uM)	C20:2 (ug/L)	0.43	0.02
Homocystein (uM)	C20:2/cre (ug/g cre)	0.40	0.03
Homocystein (uM)	C20:3 Eicosatrienoate (ug/L)	0.61	0.00
Homocystein (uM)	C20:3 Eicosatrienoate/cre (ug/g cre)	0.60	0.00
Homocystein (uM)	C20:4 (ug/L)	-0.35	0.06
Homocystein (uM)	C20:4/cre (ug/g cre)	-0.35	0.06
Homocystein (uM)	C22:1 (ug/L)	0.56	0.00
Homocystein (uM)	C22:1/cre (ug/g cre)	0.37	0.04
Homocystein (uM)	C24:0/cre (ug/g cre)	0.32	0.08
Homocystein (uM)	SAFA	0.33	0.08
Homocystein (uM)	Yogurt (g)	0.57	0.00
Kimchi (g)	C15:1 (ug/L)	0.63	0.00
Kimchi (g)	C15:1/cre (ug/g cre)	0.37	0.05
Kimchi (g)	UGT1A9	0.69	0.01
Kimchi (g)	Vegetable lipid (g)	0.42	0.02
KRAS	C22:0/cre (ug/g cre)	-0.39	0.06
KRAS	C24:0/cre (ug/g cre)	-0.37	0.08
KRAS	MGMT	-0.55	0.01
KRAS	PPARG	0.43	0.06
KRAS	SULT1A1	0.78	0.00
KRAS	TP53	0.43	0.05
LDLC (mg/dL)	C14:0 (ug/L)	0.43	0.02
LDLC (mg/dL)	C14:0/cre (ug/g cre)	0.34	0.06
LDLC (mg/dL)	C15:0 (ug/L)	0.40	0.03
LDLC (mg/dL)	C16:0 (ug/L)	0.47	0.01
LDLC (mg/dL)	C16:0/cre (ug/g cre)	0.34	0.06
LDLC (mg/dL)	C16:1 (ug/L)	0.39	0.03

LDLC (mg/dL)	C16:1/cre (ug/g cre)	0.37	0.05
LDLC (mg/dL)	C16:1T (ug/L)	0.39	0.03
LDLC (mg/dL)	C16:1T/cre (ug/g cre)	0.37	0.05
LDLC (mg/dL)	C17:0 (ug/L)	0.38	0.04
LDLC (mg/dL)	C17:0/cre (ug/g cre)	0.38	0.04
LDLC (mg/dL)	C18:0 (ug/L)	0.46	0.01
LDLC (mg/dL)	C18:0/cre (ug/g cre)	0.34	0.07
LDLC (mg/dL)	C18:1 Mix (ug/L)	0.44	0.01
LDLC (mg/dL)	C18:1 Mix/cre (ug/g cre)	0.38	0.04
LDLC (mg/dL)	C18:2 Mix (ug/L)	0.34	0.06
LDLC (mg/dL)	C18:3 Gamma Linolenate (ug/L)	0.37	0.04
LDLC (mg/dL)	C19:0 (ug/L)	0.37	0.04
LDLC (mg/dL)	C19:0/cre (ug/g cre)	0.35	0.06
LDLC (mg/dL)	C19:1 (ug/L)	0.51	0.00
LDLC (mg/dL)	C19:1/cre (ug/g cre)	0.37	0.05
LDLC (mg/dL)	C20:0 (ug/L)	0.39	0.04
LDLC (mg/dL)	C20:0/cre (ug/g cre)	0.33	0.08
LDLC (mg/dL)	C20:1 Mix (ug/L)	0.34	0.07
LDLC (mg/dL)	C20:3 Homogamma Linolenate (ug/L)	0.44	0.01
LDLC (mg/dL)	C20:3 Homogamma Linolenate/cre (ug/g cre)	0.38	0.04
LDLC (mg/dL)	C20:5 (ug/L)	0.44	0.01
LDLC (mg/dL)	C20:5/cre (ug/g cre)	0.38	0.04
LDLC (mg/dL)	C22:2 (ug/L)	0.35	0.06
LDLC (mg/dL)	dG-C8 MeIQx/1.766ug of DNA	0.44	0.02
LDLC (mg/dL)	Fruit (g)	-0.34	0.07
LDLC (mg/dL)	MUFA	0.44	0.02
LDLC (mg/dL)	MUFA/cre (ug/g cre)	0.38	0.04
LDLC (mg/dL)	NAT2	-0.55	0.03
LDLC (mg/dL)	PUFA	0.35	0.06
LDLC (mg/dL)	SAFA	0.47	0.01
LDLC (mg/dL)	SAFA/cre	0.35	0.06
LDLC (mg/dL)	total fatty acid	0.48	0.01
LDLC (mg/dL)	total fatty acid/cre	0.37	0.05
LDLC (mg/dL)	UFA (ug/L)	0.43	0.02
LDLC (mg/dL)	UFA/cre (ug/g cre)	0.36	0.05
MDA (umol/L)	1-OHP with Cre (ug/g Cre)	-0.40	0.03
MDA (umol/L)	C14:0/cre (ug/g cre)	-0.33	0.08
MDA (umol/L)	C14:1/cre (ug/g cre)	-0.35	0.05
MDA (umol/L)	C15:0/cre (ug/g cre)	-0.31	0.09
MDA (umol/L)	C15:1/cre (ug/g cre)	-0.32	0.08

MDA (umol/L)	C16:0/cre (ug/g cre)	-0.35	0.06
MDA (umol/L)	C16:1/cre (ug/g cre)	-0.31	0.09
MDA (umol/L)	C16:1T/cre (ug/g cre)	-0.31	0.09
MDA (umol/L)	C18:2 Mix/cre (ug/g cre)	-0.33	0.07
MDA (umol/L)	C18:3 Gamma Linolenate/cre (ug/g cre)	-0.31	0.09
MDA (umol/L)	C19:1/cre (ug/g cre)	-0.32	0.09
MDA (umol/L)	C20:1 Mix (ug/L)	-0.31	0.10
MDA (umol/L)	C20:1 Mix/cre (ug/g cre)	-0.35	0.06
MDA (umol/L)	C20:2/cre (ug/g cre)	-0.38	0.04
MDA (umol/L)	C20:3 Homogamma Linolenate/cre (ug/g cre)	-0.35	0.06
MDA (umol/L)	C20:5/cre (ug/g cre)	-0.34	0.07
MDA (umol/L)	C22:1 (ug/L)	-0.37	0.04
MDA (umol/L)	C22:1/cre (ug/g cre)	-0.39	0.03
MDA (umol/L)	C22:3 (ug/L)	-0.31	0.10
MDA (umol/L)	C22:3/cre (ug/g cre)	-0.36	0.05
MDA (umol/L)	C22:5 (ug/L)	0.39	0.03
MDA (umol/L)	C24:1/cre (ug/g cre)	-0.33	0.07
MDA (umol/L)	CCL2	0.94	0.00
MDA (umol/L)	MDA with Cre (uM/g cre)	0.33	0.07
MDA (umol/L)	MUFA/cre (ug/g cre)	-0.31	0.09
MDA (umol/L)	NAT2	0.62	0.01
MDA (umol/L)	PUFA/cre (ug/g cre)	-0.35	0.05
MDA (umol/L)	SAFA/cre	-0.32	0.08
MDA (umol/L)	total fatty acid/cre	-0.34	0.07
MDA (umol/L)	UFA/cre (ug/g cre)	-0.33	0.08
MDA with Cre (uM/g cre)	1-OHP (ug/L)	-0.40	0.03
MDA with Cre (uM/g cre)	1-OHP with Cre (ug/g Cre)	0.47	0.01
MDA with Cre (uM/g cre)	C14:1 (ug/L)	-0.41	0.02
MDA with Cre (uM/g cre)	C15:1 (ug/L)	-0.40	0.03
MDA with Cre (uM/g cre)	C18:1 Mix (ug/L)	-0.31	0.10
MDA with Cre (uM/g cre)	C18:2 Mix (ug/L)	-0.36	0.05
MDA with Cre (uM/g cre)	C18:3 Alpha Linolenate/cre (ug/g cre)	0.63	0.00
MDA with Cre (uM/g cre)	C18:3 Gamma Linolenate (ug/L)	-0.31	0.10
MDA with Cre (uM/g cre)	C19:1 (ug/L)	-0.37	0.04
MDA with Cre (uM/g cre)	C20:3 Homogamma Linolenate (ug/L)	-0.34	0.07
MDA with Cre (uM/g cre)	C20:4 (ug/L)	-0.43	0.02
MDA with Cre (uM/g cre)	C20:4/cre (ug/g cre)	0.37	0.05
MDA with Cre (uM/g cre)	C20:5 (ug/L)	-0.32	0.08
MDA with Cre (uM/g cre)	C22:3 (ug/L)	-0.33	0.08

MDA with Cre (uM/g cre)	C22:4/cre (ug/g cre)	0.40	0.03
MDA with Cre (uM/g cre)	C22:5/cre (ug/g cre)	0.45	0.01
MDA with Cre (uM/g cre)	C22:6 (ug/L)	-0.38	0.04
MDA with Cre (uM/g cre)	C22:6/cre (ug/g cre)	0.46	0.01
MDA with Cre (uM/g cre)	C24:0/cre (ug/g cre)	0.31	0.09
MDA with Cre (uM/g cre)	C24:1 (ug/L)	-0.38	0.04
MDA with Cre (uM/g cre)	MeIQx with Cre (ng/g cre)	0.61	0.00
MDA with Cre (uM/g cre)	MUFA	-0.31	0.09
MDA with Cre (uM/g cre)	PhIP (ng/L)	0.39	0.03
MDA with Cre (uM/g cre)	PhIP with Cre (ng/g cre)	0.62	0.00
MDA with Cre (uM/g cre)	PUFA	-0.39	0.04
MDA with Cre (uM/g cre)	UFA (ug/L)	-0.33	0.07
MDA with Cre (uM/g cre)	XPC	0.52	0.01
MeIQx (ng/L)	APC	0.62	0.00
MeIQx (ng/L)	C18:1 Mix (ug/L)	0.31	0.09
MeIQx (ng/L)	C18:2 Mix (ug/L)	0.42	0.02
MeIQx (ng/L)	C18:3 Alpha Linolenate (ug/L)	0.37	0.04
MeIQx (ng/L)	C18:3 Gamma Linolenate (ug/L)	0.43	0.02
MeIQx (ng/L)	C20:4 (ug/L)	0.43	0.02
MeIQx (ng/L)	C22:6 (ug/L)	0.43	0.02
MeIQx (ng/L)	CCL2	0.75	0.02
MeIQx (ng/L)	KRAS	0.62	0.00
MeIQx (ng/L)	MGMT	-0.41	0.05
MeIQx (ng/L)	PTGS2	0.48	0.02
MeIQx (ng/L)	PUFA	0.44	0.02
MeIQx (ng/L)	SULT1A1	0.53	0.01
MeIQx (ng/L)	UFA (ug/L)	0.34	0.07
MeIQx with Cre (ng/g cre)	C15:0/cre (ug/g cre)	0.33	0.07
MeIQx with Cre (ng/g cre)	C15:1 (ug/L)	-0.44	0.01
MeIQx with Cre (ng/g cre)	C18:0/cre (ug/g cre)	0.39	0.03
MeIQx with Cre (ng/g cre)	C18:3 Alpha Linolenate/cre (ug/g cre)	0.37	0.04
MeIQx with Cre (ng/g cre)	C20:4 (ug/L)	-0.39	0.03
MeIQx with Cre (ng/g cre)	C20:4/cre (ug/g cre)	0.54	0.00
MeIQx with Cre (ng/g cre)	C22:0/cre (ug/g cre)	0.51	0.00
MeIQx with Cre (ng/g cre)	C22:1/cre (ug/g cre)	0.65	0.00
MeIQx with Cre (ng/g cre)	C22:4 (ug/L)	-0.36	0.05
MeIQx with Cre (ng/g cre)	C22:4/cre (ug/g cre)	0.57	0.00
MeIQx with Cre (ng/g cre)	C22:5 (ug/L)	-0.35	0.05
MeIQx with Cre (ng/g cre)	C22:5/cre (ug/g cre)	0.58	0.00
MeIQx with Cre (ng/g cre)	C22:6 (ug/L)	-0.38	0.04

MeIQx with Cre (ng/g cre)	C22:6/cre (ug/g cre)	0.74	0.00
MeIQx with Cre (ng/g cre)	C24:0/cre (ug/g cre)	0.46	0.01
MeIQx with Cre (ng/g cre)	C24:1 (ug/L)	-0.33	0.07
MeIQx with Cre (ng/g cre)	C24:1/cre (ug/g cre)	0.61	0.00
MeIQx with Cre (ng/g cre)	PhIP with Cre (ng/g cre)	0.88	0.00
MeIQx with Cre (ng/g cre)	SAFA/cre	0.32	0.08
MGMT	C22:0 (ug/L)	0.46	0.03
MGMT	C24:0 (ug/L)	0.46	0.03
MGMT	SULT1A1	-0.40	0.06
MLH1	C22:1/cre (ug/g cre)	-0.35	0.10
MLH1	XPC	0.45	0.04
MUFA	MUFA/cre (ug/g cre)	0.78	0.00
MUFA	PUFA	0.88	0.00
MUFA	PUFA/cre (ug/g cre)	0.59	0.00
MUFA	UFA (ug/L)	1.00	0.00
MUFA	UFA/cre (ug/g cre)	0.75	0.00
MUFA/cre	PUFA	0.58	0.00
MUFA/cre	PUFA/cre (ug/g cre)	0.90	0.00
MUFA/cre	UFA (ug/L)	0.75	0.00
MUFA/cre	UFA/cre (ug/g cre)	1.00	0.00
NAT2	C20:4 (ug/L)	0.48	0.07
NAT2	C22:4 (ug/L)	0.56	0.03
NAT2	C22:5 (ug/L)	0.76	0.00
NAT2	C22:6 (ug/L)	0.65	0.01
PhIP (ng/L)	APC	0.52	0.01
PhIP (ng/L)	C20:4/cre (ug/g cre)	0.36	0.05
PhIP (ng/L)	C22:6/cre (ug/g cre)	0.31	0.09
PhIP (ng/L)	KRAS	0.55	0.01
PhIP (ng/L)	MeIQx with Cre (ng/g cre)	0.43	0.02
PhIP (ng/L)	PhIP with Cre (ng/g cre)	0.67	0.00
PhIP (ng/L)	PTGS2	0.51	0.01
PhIP (ng/L)	SULT1A1	0.66	0.00
PhIP (ng/L)	XPC	0.44	0.04
PhIP with Cre (ng/g cre)	C15:0/cre (ug/g cre)	0.34	0.07
PhIP with Cre (ng/g cre)	C15:1 (ug/L)	-0.38	0.04
PhIP with Cre (ng/g cre)	C18:0/cre (ug/g cre)	0.43	0.02
PhIP with Cre (ng/g cre)	C18:3 Alpha Linolenate/cre (ug/g cre)	0.39	0.04
PhIP with Cre (ng/g cre)	C19:1 (ug/L)	-0.31	0.10
PhIP with Cre (ng/g cre)	C20:4 (ug/L)	-0.36	0.05
PhIP with Cre (ng/g cre)	C20:4/cre (ug/g cre)	0.58	0.00

PhIP with Cre (ng/g cre)	C22:0/cre (ug/g cre)	0.51	0.00
PhIP with Cre (ng/g cre)	C22:1/cre (ug/g cre)	0.45	0.01
PhIP with Cre (ng/g cre)	C22:4 (ug/L)	-0.32	0.09
PhIP with Cre (ng/g cre)	C22:4/cre (ug/g cre)	0.57	0.00
PhIP with Cre (ng/g cre)	C22:5 (ug/L)	-0.33	0.08
PhIP with Cre (ng/g cre)	C22:5/cre (ug/g cre)	0.63	0.00
PhIP with Cre (ng/g cre)	C22:6 (ug/L)	-0.37	0.05
PhIP with Cre (ng/g cre)	C22:6/cre (ug/g cre)	0.75	0.00
PhIP with Cre (ng/g cre)	C24:0/cre (ug/g cre)	0.44	0.02
PhIP with Cre (ng/g cre)	C24:1 (ug/L)	-0.31	0.09
PhIP with Cre (ng/g cre)	C24:1/cre (ug/g cre)	0.60	0.00
PhIP with Cre (ng/g cre)	SAFA/cre	0.34	0.06
PhIP with Cre (ng/g cre)	total fatty acid/cre	0.32	0.08
PPARG	C14:1/cre (ug/g cre)	-0.41	0.07
PPARG	C18:3 Gamma Linolenate/cre (ug/g cre)	-0.38	0.10
PPARG	C20:5/cre (ug/g cre)	-0.42	0.07
PPARG	C22:6 (ug/L)	0.39	0.09
PPARG	C24:1 (ug/L)	0.40	0.08
Processed meat (g)	Animal lipid (g/day)	0.61	0.00
Processed meat (g)	Total lipid (g)	0.48	0.01
PTGS2	APC	0.61	0.00
PTGS2	C18:3 Gamma Linolenate (ug/L)	0.36	0.09
PTGS2	C22:6 (ug/L)	0.37	0.08
PTGS2	KRAS	0.68	0.00
PTGS2	SULT1A1	0.80	0.00
PTGS2	XPC	0.66	0.00
PUFA	PUFA/cre (ug/g cre)	0.54	0.00
PUFA	UFA (ug/L)	0.92	0.00
PUFA	UFA/cre (ug/g cre)	0.58	0.00
PUFA/cre	UFA (ug/L)	0.59	0.00
PUFA/cre	UFA/cre (ug/g cre)	0.93	0.00
Red meat (g)	C18:3 Alpha Linolenate/cre (ug/g cre)	0.36	0.06
Red meat (g)	C20:4/cre (ug/g cre)	0.40	0.03
Red meat (g)	C22:6/cre (ug/g cre)	0.40	0.03
Red meat (g)	MDA with Cre (uM/g cre)	0.43	0.02
Red meat (g)	MeIQx with Cre (ng/g cre)	0.33	0.08
Red meat (g)	PhIP with Cre (ng/g cre)	0.34	0.07
Red meat (g)	PPARG	0.43	0.06
Red meat (g)	Processed meat (g)	0.33	0.08
Red meat (g)	SULT1A1	0.37	0.09

Red meat (g)	TP53	-0.48	0.03
Red meat (g)	Animal lipid (g/day)	0.77	0.00
Red meat (g)	Total lipid (g)	0.55	0.00
SAFA	MUFA	0.82	0.00
SAFA	MUFA/cre (ug/g cre)	0.67	0.00
SAFA	PUFA	0.57	0.00
SAFA	PUFA/cre (ug/g cre)	0.43	0.02
SAFA	SAFA/cre	0.70	0.00
SAFA	UFA (ug/L)	0.78	0.00
SAFA	UFA/cre (ug/g cre)	0.64	0.00
SAFA/cre	MUFA	0.56	0.00
SAFA/cre	MUFA/cre (ug/g cre)	0.83	0.00
SAFA/cre	PUFA/cre (ug/g cre)	0.71	0.00
SAFA/cre	UFA (ug/L)	0.52	0.00
SAFA/cre	UFA/cre (ug/g cre)	0.82	0.00
TC (mg/dL)	C19:1 (ug/L)	0.32	0.09
TC (mg/dL)	C22:4 (ug/L)	-0.37	0.05
TC (mg/dL)	C22:5 (ug/L)	-0.33	0.08
TC (mg/dL)	dG-C8 MeIQx/1.766ug of DNA	0.31	0.09
TC (mg/dL)	Fruit (g)	-0.35	0.06
TC (mg/dL)	LDLC (mg/dL)	0.77	0.00
TC (mg/dL)	MDA (umol/L)	-0.31	0.10
TC (mg/dL)	MLH1	0.37	0.08
TC (mg/dL)	NAT2	-0.66	0.01
TC (mg/dL)	TG (mg/dL)	0.46	0.01
TG (mg/dL)	Processed meat (g)	0.32	0.09
TG (mg/dL)	PTGS2	-0.40	0.06
TG (mg/dL)	UGT1A9	0.46	0.10
TG (mg/dL)	Animal lipid (g/day)	0.51	0.00
TG (mg/dL)	Vegetable lipid (g)	0.51	0.00
TG (mg/dL)	Total lipid (g)	0.61	0.00
total fatty acid	MUFA	0.90	0.00
total fatty acid	MUFA/cre (ug/g cre)	0.72	0.00
total fatty acid	PUFA	0.68	0.00
total fatty acid	PUFA/cre (ug/g cre)	0.49	0.01
total fatty acid	SAFA	0.99	0.00
total fatty acid	SAFA/cre	0.68	0.00
total fatty acid	total fatty acid/cre	0.71	0.00
total fatty acid	UFA (ug/L)	0.87	0.00
total fatty acid	UFA/cre (ug/g cre)	0.69	0.00
total fatty acid/cre	MUFA	0.63	0.00

total fatty acid/cre	MUFA/cre (ug/g cre)	0.90	0.00
total fatty acid/cre	PUFA	0.36	0.05
total fatty acid/cre	PUFA/cre (ug/g cre)	0.79	0.00
total fatty acid/cre	SAFA	0.71	0.00
total fatty acid/cre	SAFA/cre	0.99	0.00
total fatty acid/cre	UFA (ug/L)	0.59	0.00
total fatty acid/cre	UFA/cre (ug/g cre)	0.89	0.00
TP53	C14:0/cre (ug/g cre)	-0.37	0.09
TP53	C15:0/cre (ug/g cre)	-0.36	0.10
TP53	C20:4/cre (ug/g cre)	-0.48	0.02
TP53	C22:0/cre (ug/g cre)	-0.40	0.07
TP53	C22:4/cre (ug/g cre)	-0.56	0.01
TP53	C22:5/cre (ug/g cre)	-0.52	0.01
TP53	C22:6/cre (ug/g cre)	-0.55	0.01
TP53	C24:1/cre (ug/g cre)	-0.52	0.01
TP53	total fatty acid/cre	-0.36	0.10
TP53	UGT1A9	0.73	0.00
UGT1A9	C15:1 (ug/L)	0.69	0.01
XPC	SULT1A1	0.42	0.05
Yogurt (g)	C14:1 (ug/L)	0.36	0.06
Yogurt (g)	C14:1/cre (ug/g cre)	0.52	0.00
Yogurt (g)	C15:0/cre (ug/g cre)	0.32	0.09
Yogurt (g)	C16:0/cre (ug/g cre)	0.35	0.06
Yogurt (g)	C16:1 (ug/L)	0.35	0.07
Yogurt (g)	C16:1/cre (ug/g cre)	0.43	0.02
Yogurt (g)	C16:1T (ug/L)	0.35	0.07
Yogurt (g)	C16:1T/cre (ug/g cre)	0.43	0.02
Yogurt (g)	C18:2 Mix/cre (ug/g cre)	0.38	0.04
Yogurt (g)	C18:3 Gamma Linolenate/cre (ug/g cre)	0.34	0.07
Yogurt (g)	C20:1 Mix (ug/L)	0.64	0.00
Yogurt (g)	C20:1 Mix/cre (ug/g cre)	0.64	0.00
Yogurt (g)	C20:2 (ug/L)	0.68	0.00
Yogurt (g)	C20:2/cre (ug/g cre)	0.56	0.00
Yogurt (g)	C20:3 Eicosatrienoate (ug/L)	0.80	0.00
Yogurt (g)	C20:3 Eicosatrienoate/cre (ug/g cre)	0.81	0.00
Yogurt (g)	C20:4/cre (ug/g cre)	-0.36	0.06
Yogurt (g)	C22:1 (ug/L)	0.66	0.00
Yogurt (g)	C22:2 (ug/L)	0.40	0.03
Yogurt (g)	C22:2/cre (ug/g cre)	0.37	0.05
Yogurt (g)	C22:3 (ug/L)	0.44	0.02

Yogurt (g)	C22:3/cre (ug/g cre)	0.52	0.00
Yogurt (g)	PUFA/cre (ug/g cre)	0.36	0.06
Animal lipid (g/day)	SULT1A1	0.37	0.09
Animal lipid (g/day)	Total lipid (g/day)	0.85	0.00
Vegetable lipid (g)	C15:1 (ug/L)	0.33	0.08
Vegetable lipid (g)	C24:1 (ug/L)	0.33	0.08
Vegetable lipid (g)	dG-C8 MeIQx/1.766ug of DNA	0.36	0.06
Vegetable lipid (g)	UGT1A9	0.69	0.01
Vegetable lipid (g)	Animal lipid (g/day)	0.41	0.03
Vegetable lipid (g)	Total lipid (g)	0.83	0.00
Total lipid (g)	UGT1A9	0.61	0.02

Table S3. MRM channels for 8 compounds including internal standards of HCAs and dG-HCAs

Compounds	Precursor ion(m/z)	Product ions(m/z)	Collision energy(eV)	Dwell time(msec)
MeIQx	214.1	199<br>131	30<br>45	20<br>20
MeIQx-d_3	217.1	199<br>131	34<br>42	20<br>20
dG-C8 MeIQx	479.1	363.1<br>299.1	17<br>64	20<br>20
dG-C8 MeIQx -d_3	482.1	366.1	17	20
PhIP	225.1	210<br>140	34<br>45	20<br>20
PhIP-d_3	228.1	210<br>140	34<br>45	20<br>20
dG-C8 PhIP	490.1	374.1<br>250.1	20<br>60	20<br>20
dG-C8 PhIP -d_3	493.1	377.1	25	20

Table S4. Rack of association between tobacco or fish intake and exposure biomarkers

Note		
DIETHABIT_06	Frequency of fish	1=rarely<br>2=one piece/2 days<br>3= ≥one piece/day
ppd	cigarette pack/day	

Variable	by Variable	Correlation	Signif Prob
PhIP with Cre (ng/g cre)	MeIQx with Cre (ng/g cre)	0.8806	0
PhIP with Cre (ng/g cre)	PhIP (ng/L)	0.669	0.0001
MeIQx with Cre (ng/g cre)	1-OHP with Cre (ug/g Cre)	0.6149	0.0003
PhIP with Cre (ng/g cre)	1-OHP with Cre (ug/g Cre)	0.5175	0.0034
MeIQx with Cre (ng/g cre)	dG-C8 MeIQx/1.766ug of DNA	-0.4278	0.0184
MeIQx with Cre (ng/g cre)	PhIP (ng/L)	0.4266	0.0187
MeIQx with Cre (ng/g cre)	1-OHP (ug/L)	-0.4183	0.0214
PhIP with Cre (ng/g cre)	1-OHP (ug/L)	-0.3462	0.061
1-OHP (ug/L)	dG-C8 MeIQx/1.766ug of DNA	0.3116	0.0937
MeIQx with Cre (ng/g cre)	MeIQx (ng/L)	0.2653	0.1566
PhIP (ng/L)	1-OHP with Cre (ug/g Cre)	0.2553	0.1734
1-OHP (ug/L)	DIETHABIT_06	-0.2353	0.2192
PhIP (ng/L)	dG-C8 MeIQx/1.766ug of DNA	-0.2301	0.2212
1-OHP with Cre (ug/g Cre)	ppd	0.2137	0.2569
1-OHP with Cre (ug/g Cre)	dG-C8 MeIQx/1.766ug of DNA	-0.19	0.3146
PhIP with Cre (ng/g cre)	DIETHABIT_06	0.1777	0.3564
MeIQx (ng/L)	1-OHP with Cre (ug/g Cre)	-0.1731	0.3602
MeIQx with Cre (ng/g cre)	DIETHABIT_06	0.1507	0.4352
dG-C8 MeIQx/1.766ug of DNA	ppd	0.1454	0.4432
C18:3 Alpha Linolenate	PhIP (ng/L)	0.142	0.454
MeIQx (ng/L)	DIETHABIT_06	-0.1301	0.5012
PhIP (ng/L)	ppd	-0.1155	0.5435
1-OHP (ug/L)	ppd	0.0935	0.6231
MeIQx (ng/L)	dG-C8 MeIQx/1.766ug of DNA	-0.0839	0.6593
PhIP (ng/L)	MeIQx (ng/L)	0.0805	0.6725
C18:3 Alpha Linolenate	1-OHP (ug/L)	-0.079	0.6781
PhIP (ng/L)	1-OHP (ug/L)	-0.0755	0.6915
C18:3 Alpha Linolenate	1-OHP with Cre (ug/g Cre)	-0.0573	0.7635
C18:3 Alpha Linolenate	DIETHABIT_06	0.0561	0.7727
ppd	DIETHABIT_06	-0.0513	0.7916
C18:3 Alpha Linolenate	ppd	-0.0412	0.8289
MeIQx with Cre (ng/g cre)	ppd	-0.0352	0.8534
PhIP with Cre (ng/g cre)	MeIQx (ng/L)	0.0307	0.8722
C18:3 Alpha Linolenate	dG-C8 MeIQx/1.766ug of DNA	0.0277	0.8843
PhIP with Cre (ng/g cre)	ppd	-0.0236	0.9015
1-OHP with Cre (ug/g Cre)	DIETHABIT_06	0.0238	0.9024

dG-C8 MeIQx/1.766ug of DNA	DIETHABIT_06	-0.0238	0.9025
MeIQx (ng/L)	ppd	-0.0223	0.9067
1-OHP with Cre (ug/g Cre)	1-OHP (ug/L)	-0.0218	0.9091
MeIQx (ng/L)	1-OHP (ug/L)	0.0089	0.9626
PhIP (ng/L)	DIETHABIT_06	-0.0006	0.9974

Table. S5. Sequences of the sense and antisense primers used for quantitative real time PCR

Gene	Sense primer	Antisence primer
CCL2	AGAAGAATCACCAGCAGCAAG	GTCTTCGGAGTTTGGGTTTG
PTGS2	GCCATACAGCAAATCCTTGC	TCCATAGAATCCTGTCCGGG
APC	TGCCAGTAAATAAAAGTGCTATGAC	AAATTGAAGTGTTTACAAAGTGGTG
KRAS	CTGTGTCCCCACGGTCATC	GCTCTTGATTTGTCAGCAGGA
MLH1	TCAGGTTATCGGAGCCAGC	GCAAGCATCTCAGCCTTCTT
TP53	TGCTCAGATAGCGATGGTCT	CACCACCACACTATGTCGAA
XPC	AGGACACACACAAGGTTCAC	CAGCACTCTGGTAAAGCGG
LEP	AACCCTGTGCGGATTCTTG	GACTGCGTGTGTGAAATGTC
PPARG	TCAGAAATGCCTTGCAGTGG	GATCTCCGCCAACAGCTTC
APOA1	GCCCTACCTGGACGACTTC	CTCAGCTTCTCTTGCAGCTC
MGMT	TTTCCAGCAAGAGTCGTTCA	ATGAGGATGGGGACAGGATT
CYP1A2	TCCCTGAGAGTAGCGATGAG	TAGGCAGGTAGCGAAGGATG
SULT1A1	CGTGGACTTCGTGGTTCAG	GTGAAGGTGGTCTTCCAGTC
NAT2	TACTGTTTGGTGGGCTTCATC	TCTTCAACCTCTTCCTCAGTG
UGT1A9	TGGAAAGCACAAGTACGAAG	GGATCGAGAAACACTGCATC
RPLP0	ACTGCTGCCTCATATCCGGG	GCAGCTGGCACCTTATTGGC
